# Hemochromatosis, alcoholism and unhealthy dietary fat: a case report 

**DOI:** 10.1186/s13256-020-02610-7

**Published:** 2021-02-17

**Authors:** Venkatachalam Shobi, Awe Adeseye, Ballard Billy, Kalliny Medhat

**Affiliations:** 1 Internal Medicine, Meharry Medical College, Nashville, USA; 2 Oral and Maxillofacial Surgery, Meharry Medical College, Nashville, USA; 3 Pathology, Meharry Medical College, Nashville, USA; 4 Family Medicine, Meharry Medical College, Nashville, USA; 5 Meharry Medical College, Nashville, USA

**Keywords:** Alcoholism, Unhealthy diet, Hereditary hemochromatosis, Case report

## Abstract

**Background:**

Hereditary hemochromatosis is an autosomal recessive disorder where the clinical phenotype of skin pigmentation and organ damage occurs only in homozygotes. Simple heterozygotes, that is, just C282Y, typically do not develop iron overload. Here we present a case where a simple heterozygote in combination with alcoholism developed high ferritin and high transferrin saturation levels indicative of iron overload. Though alcoholism alone could explain her presentation, we hypothesize that an inflammatory cocktail of iron and alcohol probably caused our patient to succumb to acute liver failure at a very young age.

**Case presentation:**

A 29-year-old Caucasian woman presented to the hospital with progressively worsening yellowish discoloration of her eyes and skin associated with anorexia, nausea, vomiting, diffuse abdominal discomfort, increasing abdominal girth, dark urine and pale stools for about 2 weeks. Family history was significant for hereditary hemochromatosis. Her father was a simple heterozygote and her grandmother was homozygous for C282Y. Physical examination showed scleral icterus, distended abdomen with hepatomegaly and mild generalized tenderness. Lab test results showed an elevated white blood cell count, ferritin 539 ng/dL, transferrin saturation 58.23%, elevated liver enzymes, elevated international normalized ratio (INR), low albumin, Alcoholic Liver Disease/Nonalcoholic Fatty Liver Disease (ALD/NAFLD) Index (ANI) of 2.6, suggesting a 93.2% probability of alcoholic liver disease, and phosphatidyl ethanol level of 537ng/ml. Genetic testing showed that the patient was heterozygous for human homeostatic iron regulator protein (HFE) C282Y mutation and the normal allele. Computed tomography (CT) of the abdomen revealed hepatomegaly, portal hypertension and generalized anasarca. Magnetic resonance cholangiopancreatography (MRCP) showed negative results for bile duct pathology. Workup for other causes of liver disease was negative. A diagnosis of acute alcoholic hepatitis was made, with Maddrey’s discriminant function of > 32, so prednisolone was started. Her bilirubin and INR continued to increase despite steroids, and the patient unfortunately died.

**Conclusion:**

Our case highlights the importance of considering hemochromatosis in the differential diagnosis of young patients presenting with liver failure, including cases suggestive of alcoholism as the likely etiology. Larger studies are needed to investigate the role of non-iron factors like alcohol and viral hepatitis in the progression of liver disease in simple heterozygotes with hereditary hemochromatosis, given the high prevalence of this mutation in persons of Northern European descent.

## Background

**Fig. 1:**
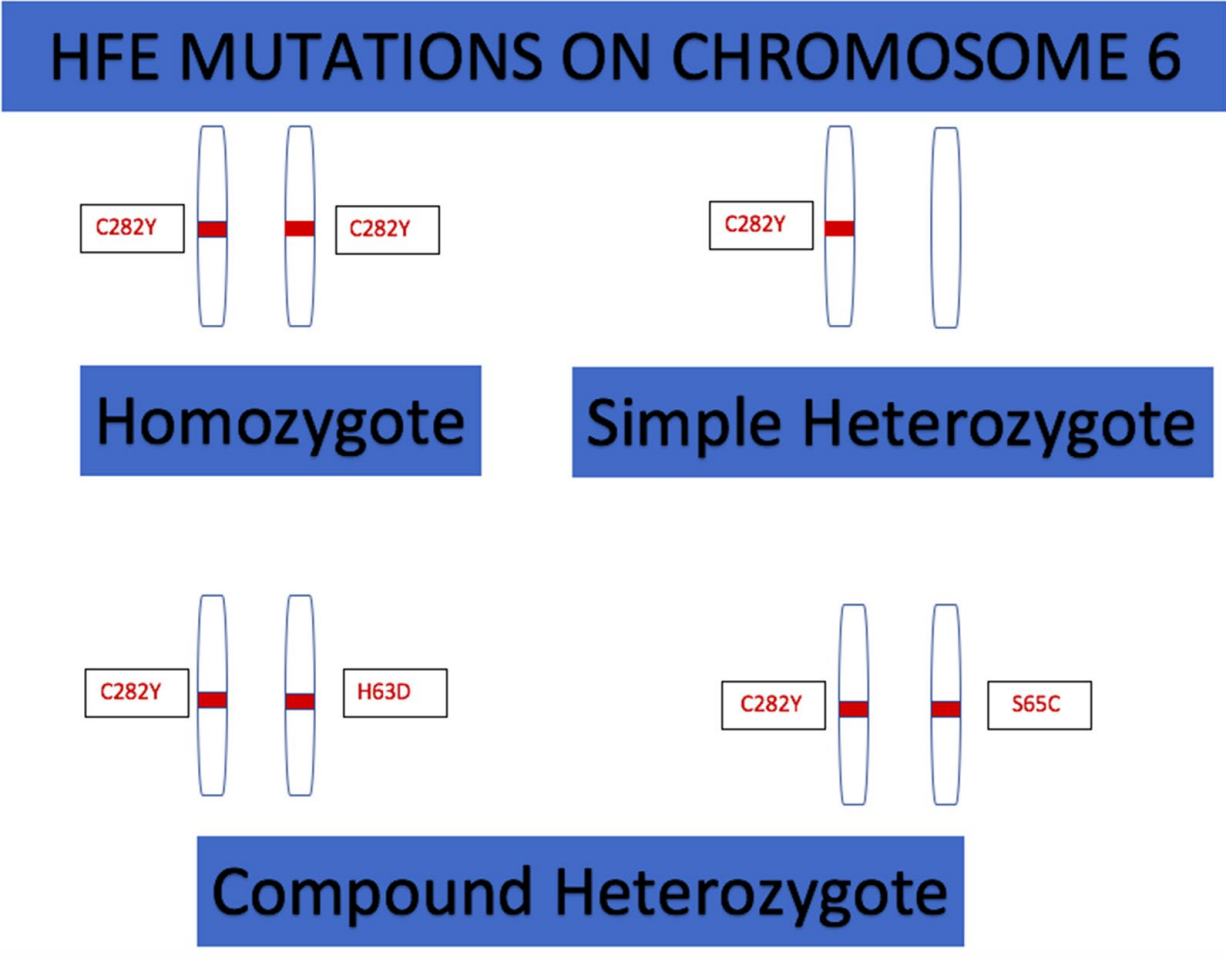
HFE gene mutations causing Hereditary Hemochromatosis

Hereditary hemochromatosis (HH) (Fig. 1) is an autosomal recessive disorder where the clinical phenotype of skin pigmentation and organ damage occurs only in homozygotes; even in homozygotes, the phenotype has a broad spectrum depending on sex and penetrance which is age-related [1, 2]. With regard to heterozygotes, it is mostly the compound heterozygotes—C282Y and the H63D or S65C variant allele—that develop iron overload [3, 4]. Simple heterozygotes—that is, just C282Y—almost never develop iron overload or organ damage [5]. Although the role of non-iron-related factors like alcohol in modulating the iron threshold required to induce liver damage is well known, the strength of their association in each of the HH phenotypes remains an area that is largely unexplored.

We present a case where a simple heterozygote with alcoholism developed high ferritin and high transferrin saturation indicative of iron overload. This is very rare considering the young age, female sex and the genotype of the patient. The iron overload coupled with probable unhealthy dietary habits in the setting of alcoholism (more fat, less essential nutrients as reported in studies) [6] resulted in an inflammatory cocktail and caused our patient to succumb to acute liver failure at a young age.

## Case presentation

A 29-year-old Caucasian woman presented to the hospital with 2 weeks of progressively worsening yellowish discoloration of her eyes and skin associated with anorexia, nausea, vomiting, diffuse abdominal discomfort, increasing abdominal girth, dark urine and pale stools. Past medical history was significant for prior episodes of hospitalization for acute alcoholic intoxication including an episode a few months prior. Imaging at that time showed hepatic steatosis but no features suggestive of hepatic cirrhosis or portal hypertension. Family history was significant for hereditary hemochromatosis. The patient’s father was heterozygous for C282Y and the paternal grandmother was homozygous for C282Y. The patient reported drinking about 1–2 glasses of wine every day, and denied smoking and illicit drug use. Vitals signs were as follows: pulse rate 94 beats per minute, respiratory rate 20 per minute, blood pressure 112/78 mmHg, temperature 36.9 °C and oxygen saturation 100% on room air. Physical examination showed scleral icterus, distended abdomen with hepatomegaly and mild generalized tenderness.

**Fig. 2:**
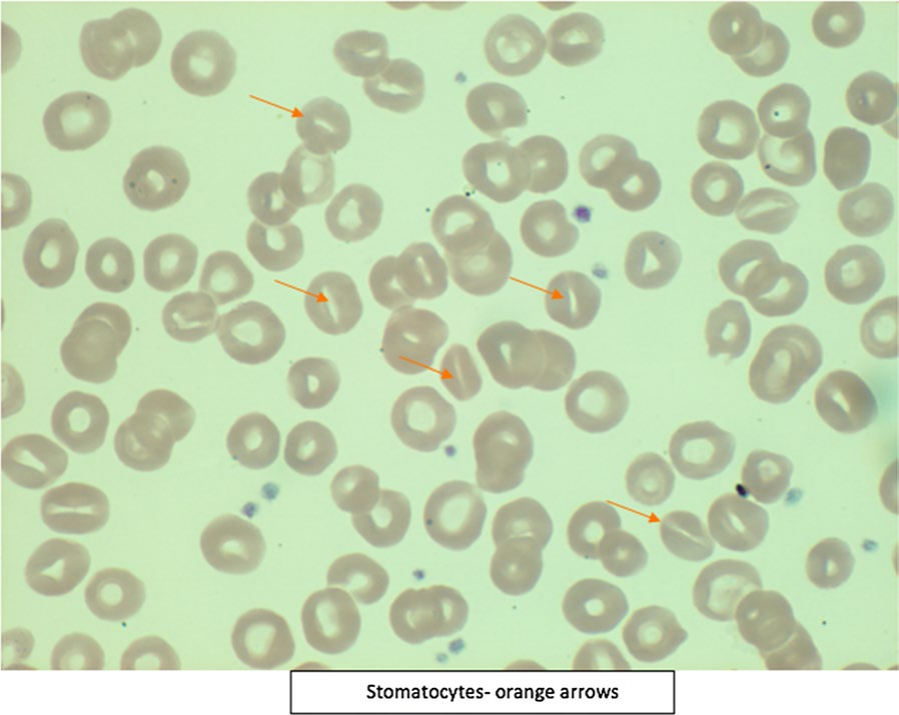
Peripheral smear showing stomatocytosis (orange arrows pointing to the stomatocytes)

**Fig. 3:**
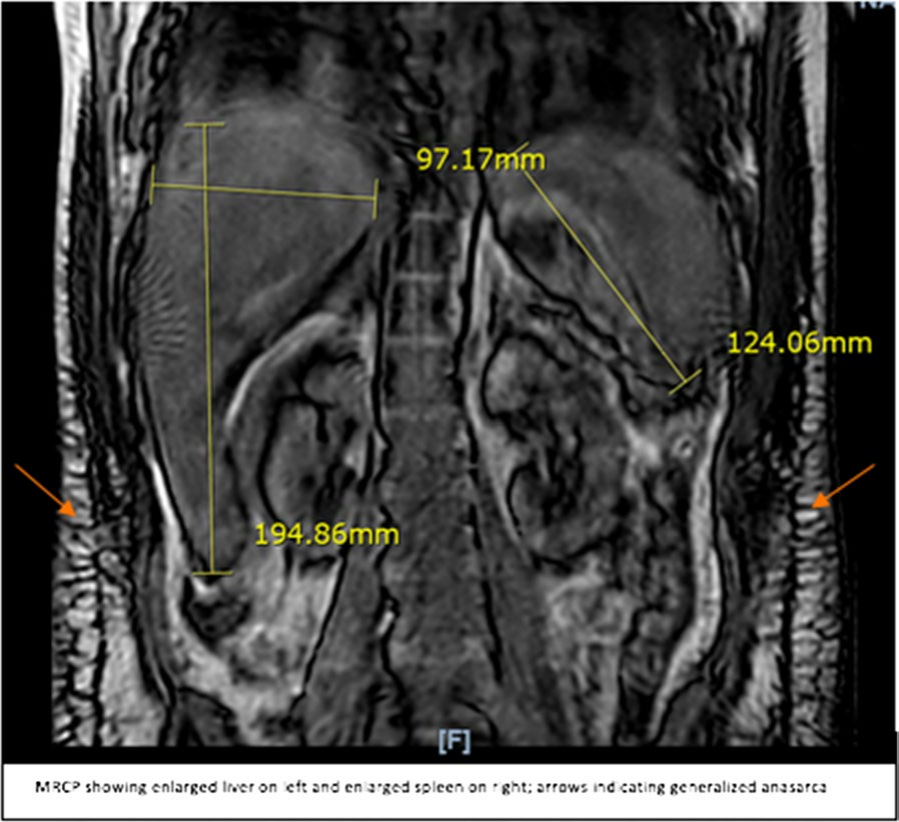
Magnetic Resonance Cholangiopancreatography (MRCP) showing enlarged liver on the left side of the image (shown using yellow array of lines on the left), enlarged spleen on the right side of the image (shown using yellow array of lines on the right) and generalized anasarca (pointed using orange arrows)

Laboratory results were as follows (Table 1): white blood cell count 31,600/μL, hemoglobin 10.1 g/dL, platelets 172/μL, ferritin 539 ng/dL, transferrin saturation 58.23%, peripheral blood smear showing stomatocytosis (Fig. 2), total bilirubin 8.7 mg/dL, direct bilirubin 7.4 mg/dL, aspartate aminotransferase (AST) 90 U/L, alanine aminotransferase (ALT) 30 U/L, alkaline phosphatase (ALKP) 420 U/L, prothrombin time (PT) 18.6 seconds, international normalized ratio (INR) 1.5, blood urea nitrogen 1.0 mg/dL, serum creatinine 0.5 mg/dL, Alcoholic Liver Disease/Nonalcoholic Fatty Liver Disease (ALD/NAFLD) Index (ANI) 2.6, suggesting a 93.2% probability of alcoholic liver disease, and phosphatidyl ethanol level 537 ng/ml (levels > 20 ng/ml indicate moderate–heavy ethanol consumption). Genetic testing results revealed that the patient was heterozygous for the HFE C282Y mutation and the normal allele, as well as negative for H63D and S65C. Imaging showed features suggestive of parenchymal liver disease, portal hypertension and generalized anasarca (Fig. 3). Magnetic resonance cholangiopancreatography (MRCP) was negative for bile duct pathology. Workup for other causes of liver disease including autoimmune hepatitis, Wilson’s disease, alpha-1 antitrypsin deficiency, celiac disease, primary biliary cirrhosis (PBC), Epstein–Barr virus (EBV), cytomegalovirus (CMV), viral hepatitis, tick-borne illnesses and leptospirosis all returned negative results. Biopsy of the liver was considered but was held due to the patient’s worsening general medical condition.

**Table 1:** Trend of laboratory values over the course of hospitalization

Laboratory test	Day 1	Day 8	Day 16	Day 23	Reference range with units
WBC	31.6	50.5	84	48.6	4.5–10.0 10 × 3/μL
RBC	3.08	2.95	2.64	2.10	4.00–5.00 10 × 6/μL
Platelets	172	370	309	176	140–400 10 × /μL
Hemoglobin	10.1	9.6	8.7	7.0	12.0–16.0 g/dL
Hematocrit	27.9	29.3	27.2	20.3	39.0–54.0%
MCV	90.6	99.3	103	96.7	80–99 fl
MCH	32.8	32.5	33	33.3	27–34 pg
MCHC	36.2	32.8	32	34.5	32–36 g/dL
RDW	18.1	23.2	24.6	22.8	39.0–54.0%
INR	1.5	2.2	1.3	1.9	0.1–1.4
BUN	1	5	38	104	7–17 mg/dL
Creatinine	0.55	0.53	0.73	4.28	0.55–1.02 mg/dL
Total bilirubin	8.7	10.3	17.4	24.7	0.2–1.3 mg/dL
Direct bilirubin	7.4		11.3		0.0–0.2 mg/dL
AST	90	64	45	71	15.00–37.00 U/L
ALT	30	23	27	12	12–78 U/L
ALKP	420	383	245	168	46–116 U/L

*WBC* white blood cells, *RBC* red blood cells, *MCV* cytomegalovirus, *MCHC* mean corpuscular hemoglobin concentration, *RDW* red cell distribution width, *BUN* blood urea nitrogen, *AST* aspartate aminotransferase, *ALT* alanine aminotransferase, *ALKP* alkaline phosphatase

The patient was diagnosed with acute alcoholic hepatitis, and Gastroenterology was consulted. The patient’s Maddrey’s discriminant function was 46.4 (a score > 32 indicates poor prognosis and that the patient might benefit from glucocorticoid therapy), so orally administered prednisolone 40 mg/day was started. Although the patient’s total bilirubin (TBIL) and INR initially improved after initiating steroidal therapy, a rebound increase was noted (Table 1), raising concerns for impending liver failure. Also, her creatinine increased from 0.5 to 4 mg/dL. Nephrology was consulted, and a diagnosis of hepatorenal syndrome type 1 was favored. In addition to worsening TBIL, INR and creatinine, the patient developed encephalopathy, succumbed to the disease and died.

## Discussion

In this article we focus on the mechanisms of liver injury from the effects of iron, alcohol and dietary habits, and it may not be surprising to see that some of these mechanisms overlap.

### Mechanism of liver injury with iron

Excess iron in the hepatocytes and Kupffer cells results in the Fenton reaction and reactive oxygen species production. The free radicals induce lipid peroxidation, which damages the mitochondria, resulting in release of cytochrome c and liver cell apoptosis. Iron overload also stimulates the production of proinflammatory and pro-fibrogenic cytokines including transforming growth factor beta (TGF-β). TGF-β leads to the activation of hepatic stellate cells and excess collagen production. Excess collagen and cross-linking coupled with iron inhibit activation of the liver progenitor cells required for the regeneration of liver cells, resulting in fibrosis [2, 7].

### Mechanism of liver injury with alcohol

Alcohol is metabolized to acetaldehyde. Acetaldehyde results in the generation of reactive oxygen species, which causes lipid peroxidation and cell membrane and DNA damage. Damaged hepatocytes express antigens which are otherwise hidden from the immune system, resulting in immune stimulation. Chronically heightened immune activity results in immune exhaustion, overwhelming bacterial infection, multi-organ damage and death. Also, chronic alcohol abuse results in overgrowth of gut bacteria, and this along with alcohol-induced leaky gut results in increased delivery of endotoxins to the liver and liver damage [8].

### Mechanism of liver injury with non-healthy dietary habits

Excess dietary fat increases insulin resistance and hyperinsulinemia, which leads to accumulation of fatty acids. Accumulated fatty acids result in the generation of lipotoxic species, hepatocellular oxidant stress and cell death. The dying hepatocytes release signals and express antigens which are otherwise hidden from the immune system. turning on the immunogenic and fibrogenesis cascade [9]. Individual susceptibility to fatty acid-induced oxidant stress depends on other factors including iron overload states such as HFE and alcoholism [2].

**Fig. 4:**
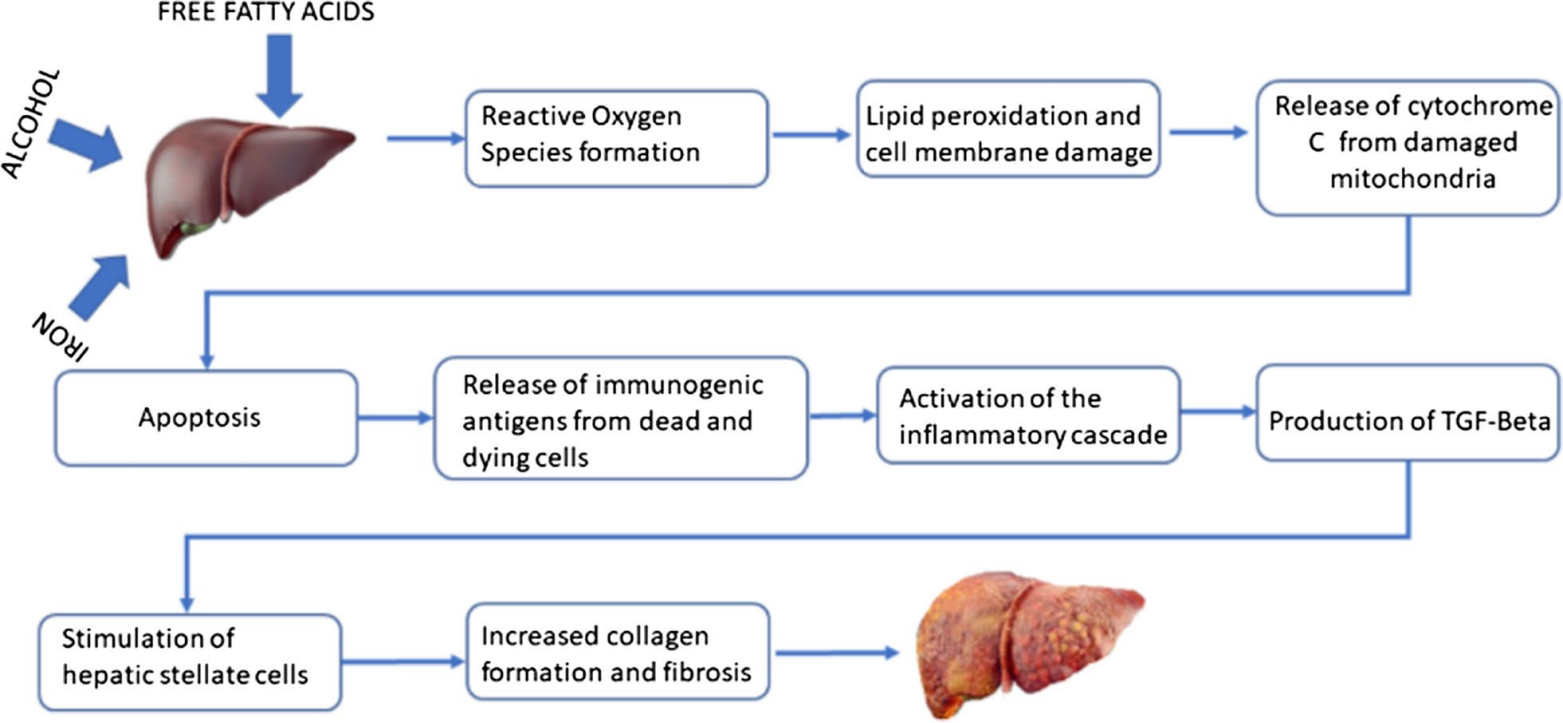
Mechanisms of liver injury from the effects of iron, alcohol and dietary habits

Thus, many of the mechanisms of liver injury from iron, alcohol and unhealthy dietary habits overlap (Fig. 4). Although non-HH factors like alcoholism, NAFLD and nonalcoholic steatohepatitis (NASH) are associated with hyperferritinemia from chronic inflammation, the patient’s elevated transferrin saturation [(serum iron/total iron binding capacity) × 100] can only be explained by her HH status. While heavy alcohol consumption alone could cause severe liver damage, we hypothesize that her HFE status and possible unhealthy dietary fat in the setting of alcoholism accelerated the progression of liver disease. Studies have shown that alcoholics consume a higher amount of fatty food and carbohydrates along with lower consumption of vegetables and dairy products, which could have a detrimental effect on health [6].

Clinicians must continually probe for factors like personal or family history of hemochromatosis, dietary habits and alcoholism using different strategies and reformatting questions. This is especially important because early recognition followed by referral to specialized centers for treatment for hereditary hemochromatosis and detoxification would be pivotal in the prognosis of these patients. Our patient persistently denied any unhealthy alcohol use until later in the disease course. This, coupled with her blood alcohol level of < 3 at admission, very high white blood cell counts, young age and female sex, pointed more towards other differentials like autoimmune hepatitis and infectious etiologies. Although we were fortunate enough to be redirected towards alcohol as the etiology from reports of stomatocytosis in the peripheral blood, high ANI and very high phosphatidyl ethanol level, the patient unfortunately succumbed to her acute liver failure.

## Conclusion

Considering the high prevalence of HH and the rising mortality from alcoholic liver disease among young adults [10, 11], more studies exploring the role of alcohol in the development of liver damage in simple heterozygotes and vice versa are essential to determine whether all alcoholics have to be screened for hereditary hemochromatosis. This is because more than one factor may often be involved in the pathogenesis and progression of liver dysfunction.

## Abbreviations

WBC: White blood cells
TS: Transferrin saturation [(serum iron/total iron binding capacity) × 100]
ALT: Alanine transaminase
AST: Aspartate transaminase
ALKP: Alkaline phosphatase
PT: Prothrombin time
INR: International normalized ratio
ALD: Alcoholic liver disease
NAFLD: Nonalcoholic fatty liver disease
ANI: ALD/NAFLD Index
CT: Computed tomography
MRCP: Magnetic resonance cholangiopancreatography
HFE: Human homeostatic iron regulator protein
HH: Hereditary hemochromatosis
TGF: Transforming growth factor
TBIL: Total bilirubin
GI: Gastroenterology
NASH: Nonalcoholic steatohepatitis
PBC: Primary biliary cirrhosis

## Availability of data and material

Our patient’s health record is available in our electronic medical record system, and information can be verified if required by the reviewer.
